# Design and Analysis of a Cardioid Flow Tube Valveless Piezoelectric Pump for Medical Applications

**DOI:** 10.3390/s24010122

**Published:** 2023-12-25

**Authors:** Jialong Wang, Fan Zhang, Zhenzhen Gui, Yuxin Wen, Yaohua Zeng, Tang Xie, Tian Tan, Bochuan Chen, Jianhui Zhang

**Affiliations:** School of Mechanical and Electrical Engineering, Guangzhou University, Guangzhou 510006, China; 2112107004@e.gzhu.edu.cn (J.W.); zhenzhengui@gzhu.edu.cn (Z.G.);

**Keywords:** piezoelectric pump, valveless, CFD simulation, cardioid tube, secondary flow

## Abstract

Piezoelectric pumps play an important role in modern medical technology. To improve the flow rate of valveless piezoelectric pumps with flow tube structures and promote the miniaturization and integration of their designs, a cardioid flow tube valveless piezoelectric pump (CFTVPP) is proposed in this study. The symmetric dual-bend tube design of CFTVPP holds great potential in applications such as fluid mixing and heat dissipation systems. The structure and working principle of the CFTVPP are analyzed, and flow resistance and velocity equations are established. Furthermore, the flow characteristics of the cardioid flow tube (CFT) are investigated through computational fluid dynamics, and the output performance of valveless piezoelectric pumps with different bend radii is studied. Experimental results demonstrate that CFTVPP exhibits the pumping effect, with a maximum vibration amplitude of 182.5 μm (at 22 Hz, 100 V) and a maximum output flow rate of 5.69 mL/min (at 25 Hz, 100 V). The results indicate that a smaller bend radius of the converging bend leads to a higher output flow rate, while the performance of valveless piezoelectric pumps with different diverging bends shows insignificant differences. The CFTVPP offers advantages such as a high output flow rate, low cost, small size for easy integration, and ease of manufacturing.

## 1. Introduction

With the rapid development of microelectronics and micromechanical technologies, miniaturized, integrated, and intelligent devices are increasingly being applied in various fields [1]. These devices require precise control and manipulation of fluids on microliter and nanoliter scales and need to enable regulation of the output flow rate on microscopic scales [2,3,4,5]. Therefore, microfluidic pumping devices have become a hot topic in scientific and technological research. Among many microfluidic pumping devices, piezoelectric pumps have received widespread attention and research, given their inherent advantages. The key component of a piezoelectric pump is the piezoelectric material, which undergoes deformation due to the inverse piezoelectric effect when an alternating electric field is applied, resulting in vibrations at the same frequency as the electric field. These vibrations cause periodic compression and expansion of the pump chamber, leading to controlled fluid flow within the pump. Owing to their unique working principle, piezoelectric pumps have the characteristic of fast response [6,7,8,9,10]. They can quickly switch between different operating states and achieve instantaneous fluid control. This characteristic is crucial in applications requiring rapid response and real-time adjustment, with broad prospects in microfluidic transport fields such as medical microinfusion [11,12,13,14,15,16,17,18], fuel cells [19,20,21,22,23,24], cooling systems [25,26,27,28,29], micromanipulation [30,31], and surface coating of fan blades [32]. In recent decades, researchers have extensively studied piezoelectric pumps and incorporated them into their products. For example, in 2008, Ma et al. integrated an air pump into an air-fed proton exchange membrane fuel cell system using a piezoelectric driving structure. When the piezoelectric oscillator moved outward, it increased the volume of the cathode channel, allowing air to be drawn into the chamber. Conversely, inward movement reduced the channel volume, compressing air into the catalyst layer and enhancing the chemical reaction. Ultimately, the hydrogen gas inlet velocity reached 0.25 m/s under optimal operating conditions [33]. In 2021, You et al. proposed a wireless micropump based on acoustic streaming for portable drug delivery systems, with a key component being a gigahertz surface-mounted resonator (SMR). The dimensions of the micropump were 9 mm × 9 mm × 9 mm. Through the inverse piezoelectric effect, the SMR generated thickness-extensional mode acoustic waves by applying voltage to the top and bottom electrodes. When in contact with the liquid, the acoustic waves propagated into the liquid, generating streaming and continuous flows and achieving a maximum flow rate of 1.34 mL/min [34]. In 2023, Fan et al. developed a jet impingement cooling system driven by an integrated piezoelectric pump. The overall size of the pump was 40 mm × 40 mm × 10 mm, and its pump chamber featured a distributed return flow with jet arrays for impingement cooling. The coolant was drawn into the chamber through an inlet and nozzle and sprayed onto the cooling surface to remove heat. Under the operation of an external water tank, the flow rate reached 306 mL/min [35].

From the aforementioned studies, while piezoelectric pumps play a role in various fields, the incorporation of internal valves increases the size and structural complexity, and compression of the volume hinders the improvement of flow velocity. Simultaneously meeting the requirements of high output flow rate, high output pressure, and small size is difficult, making their further miniaturization and integration in the field of microfluidic transport challenging. Therefore, many researchers have delved into the structural design of pump bodies. Valveless piezoelectric pumps utilizing the difference in forward and reverse flow resistances to achieve valve functions represent a promising research direction. In 2010, Wang et al. proposed a valveless piezoelectric pump with an acoustic functional pumping chamber. The pump consisted of a nozzle-shaped driving chamber with an acoustic resonance profile, serving as a pumping chamber and a flow-rectifying structure. It operated stably at driving frequencies below 100 Hz, making it suitable for microfluidic integrated systems [36]. In 2013, Pei et al. introduced a novel bidirectional pumping principle based on a dynamic rectification mechanism. The pump comprised a movable actuating platform and a gourd-shaped pumping chamber, in which the gourd-shaped chamber functioned as a rectifying structure and a pumping structure. Asymmetric deflection of the thin diaphragm generated a pressure difference in the chamber, resulting in pumping effects with maximum forward and reverse flow velocities of 1.52 and 1.48 mL/min, respectively [37]. In 2020, Zhang et al. designed a valveless piezoelectric pump with a fluid-guiding body and dual outlets. The pump chamber incorporated a streamlined fluid-guiding structure and a dual-outlet configuration, reducing the energy of the backflowing liquid [37]. In 2022, Liu et al. proposed a valveless piezoelectric pump with multistage mixing characteristics. The pump integrated two pump chambers, which were connected by a composite flow tube composed of diffuser/nozzle tubes and Tesla tubes. These combined fluid transport and mixing functions were utilized for the controlled synthesis of silver nanoparticles [38].

Based on previous research on piezoelectric pump-driven fluid transport, this paper presents a valveless piezoelectric pump consisting of a pair of cardioid flow tubes (CFTs) arranged in a horizontal plane with a vertical offset. The valveless piezoelectric pump achieves efficient fluid transport while offering potential applications in fluid mixing and heat dissipation systems, making it a valuable reference for valveless piezoelectric pump design in these fields. Five different CFT structures were designed to investigate the relationship between fluid pressure loss and tube size parameters. Subsequently, experimental prototypes of the CFT-based valveless piezoelectric pump were manufactured, and theoretical, simulated, and experimental approaches were applied to demonstrate the flow resistance characteristics and pumping effect of the CFT-based valveless piezoelectric pump. The output performance of the five valveless piezoelectric pumps was analyzed. The manufactured pump prototypes had a dimension of 45 mm × 35 mm × 8 mm, with a maximum output flow rate of 5.69 mL/min. The valveless piezoelectric pump offers advantages such as high output flow rates, low cost, small size for easy integration, and ease of manufacturing.

## 2. Design and Working Principle

### 2.1. Structural Design of CFT and CFT Valveless Piezoelectric Pump (CFTVPP)

**Figure 1:**
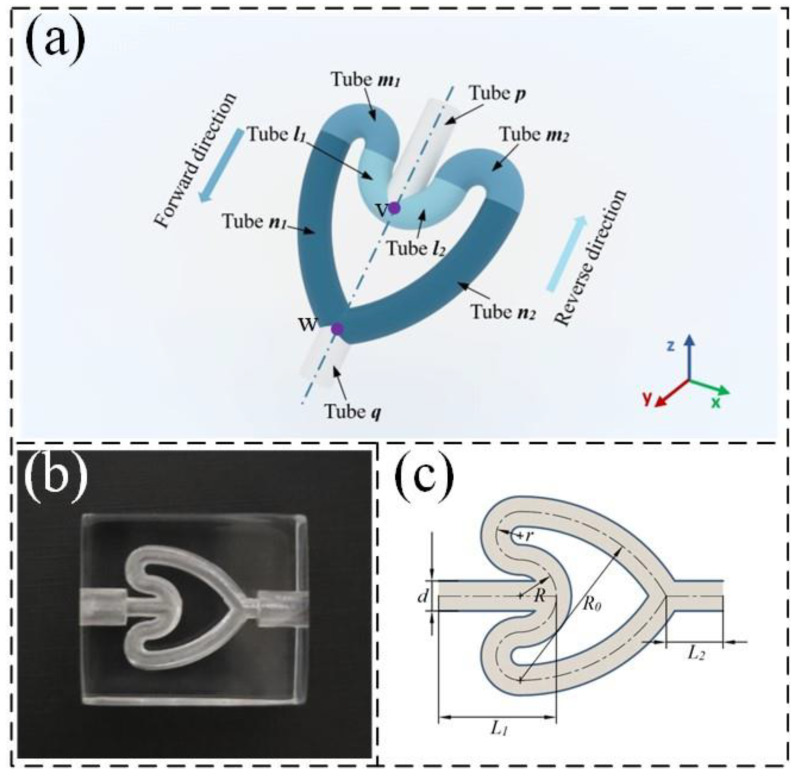
CFT structure. (**a**) Three-dimensional structure; (**b**) physical drawing of the CFT; (**c**) two-dimensional structure of the CFT.

As shown in Figure 1, the CFT is composed of four parts. Tubes *p* and *q* are the collecting tubes, with lengths L_1_ and L_2_ of 8 and 4 mm, respectively. Tubes *l*_1_, *l*_2_, *m*_1_, *m*_2_, *n*_1_, and *n*_2_ are branching tubes consisting of different arc segments. They are symmetrically distributed along the centerlines of the collecting tubes *p* and *q*. The subscripts 1 and 2 represent the left and right-branching tubes, respectively. The fluid flows into tube *p*, branches at the concave bifurcation point v, and passes through tubes *l*, *m*, and *n* before merging into tube *q* at the convex bifurcation point w. This direction is defined as the forward flow. The opposite direction is defined as the reverse flow. Tubes *l*, *m*, and *n* have curved sections with radii of curvature R, r, and R_0_, respectively. All tubes have a diameter of 2.5 mm, R_0_ is 14 mm, and the arc segments of the tubes are tangentially connected. The overall shape of the flow tube resembles a cardioid shape. Tubes *l* and *m* are the main curved sections of the CFT and serve two main purposes: creating a difference in flow resistance between the forward and reverse flows and limiting backflow to improve pumping efficiency. These purposes can be explained by the momentum theorem and the energy dissipation from fluid–solid collisions. First, when the fluid in the branching tubes converges at point v, the flow directions on both sides are opposite. At point w, the flow directions on both sides form an angle. Therefore, the momentum loss from the reverse flow is greater. Second, compared with that for the forward flow, the distance from the bifurcation point w to the fluid–solid collision point is longer than the distance from the bifurcation point v to the fluid–solid collision point for the reverse flow. Consequently, because of inertia, the energy loss from the reverse flow is greater. Based on this analysis, the CFT can be used as a valve component. The radius of curvature of the curved tubes determines the difference in resistance between forward and reverse flows, directly affecting the output performance of the CFTVPP. Therefore, we designed five different CFTs by changing the radius of curvature of the curved tubes. The specific parameters are listed in Table 1.

**Figure 2:**
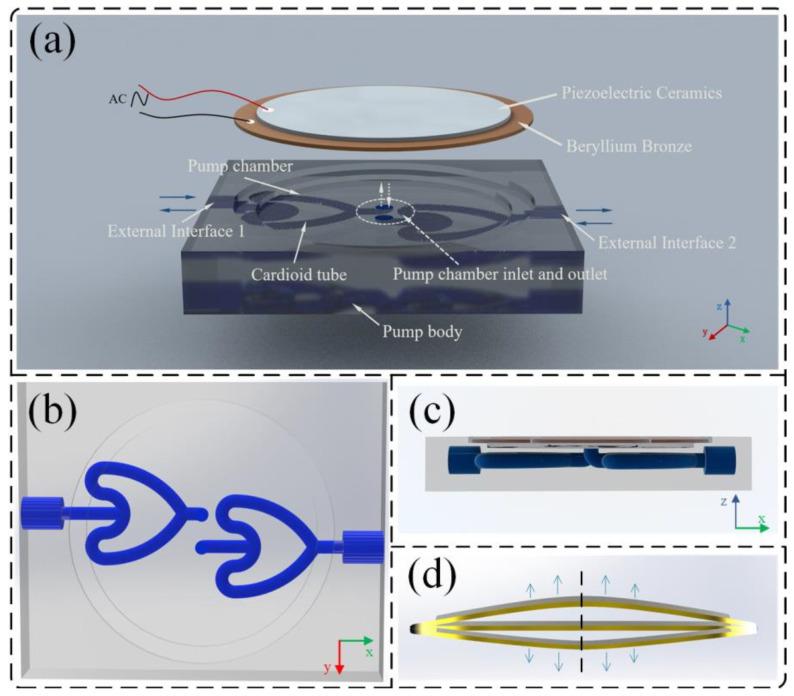
Schematic structure of CFTVPP. (**a**) assembly diagram of CFTVPP; (**b**) top view of CFTVPP; (**c**) front view of CFTVPP; (**d**) schematic of piezoelectric vibrator vibration.

**Figure 3:**
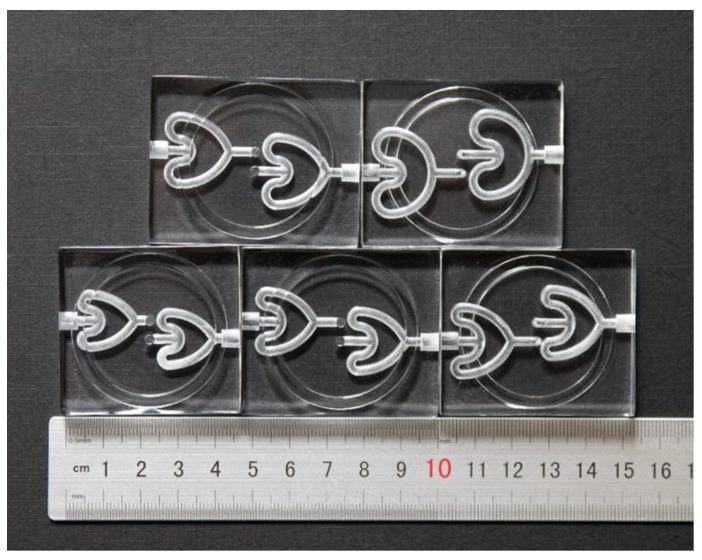
Dimensional parameters of five CFTs.

Figure 2 illustrates the structure of the CFTVPP proposed in this study. The pump consists mainly of a PZT vibrator and a pump body. The PZT vibrator is composed of a 35 mm-diameter circular piezoelectric ceramic plate and a 41 mm-diameter circular metal plate, which are bonded together. While providing sealing for the pump chamber, the vibrator undergoes periodic bending deformation when excited by a sinusoidal signal. The diameter of the pump chamber is 35 mm, and its height is 0.8 mm. The central part of the bottom surface of the pump chamber has two inlet and outlet ports, which are connected to the CFT through a quarter-circle curved tube. The distance between the inlet/outlet ports and the center of the chamber is 2.5 mm. The radius of the curved tube is 1.5 mm. The CFTs are placed horizontally, with their respective symmetric axes parallel to the edge of the pump body, and they are externally connected through interfaces. The final dimensions of the CFTVPP prototype are 45 mm × 35 mm × 8 mm. The size parameters of the pump body are shown in Figure 3. The PZT vibrator (Dongguan Kesen Electrical Co., Ltd., Dongguan, China) used in this study has a resonant impedance of 250 Ω and a resonant frequency of 2.6 ± 0.5 kHz. Its structural parameters are listed in Table 2.

### 2.2. Working Principle of CFTVPP

**Figure 4:**
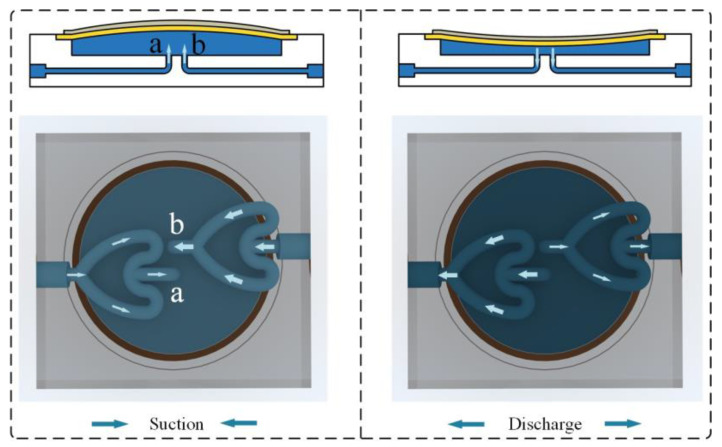
CFTVPP working principle diagram.

In the operating state, the vibration of the piezoelectric vibrator causes the liquid in the pump chamber to be periodically sucked in and discharged through the connecting holes at the bottom of the pump chamber and the two CFTs. To ensure one-way flow, it is sufficient to connect the two CFTs to the pump chamber through different ports. Considering the overall regularity and better integration into the pump body, in CFTVPP, the CFTs are assembled in the same direction, causing the fluid to flow through the tubes in opposite directions at the inlet and outlet of the pump chamber. Figure 4 illustrates the working principle of CFTVPP, with the inlet and outlet labeled as a and b, respectively. Driven by an AC voltage, the piezoelectric vibrator undergoes vertical oscillations, resulting in periodic variations in the pump chamber volume. When the piezoelectric vibrator moves upward, the volume of the pump chamber increases, leading to a decrease in pressure. This phenomenon causes the fluid to be drawn into the pump chamber through the CFTs, with one side experiencing forward flow and the other side experiencing backward flow. Conversely, when the piezoelectric vibrator moves downward, the fluid is squeezed out of the pump chamber through the tubes, resulting in a reversal of the flow direction. Owing to the asymmetrical fluid flow resistances in the tubes, the fluid flow rate through the CFTs is higher in the forward direction than in the backward direction. Under the excitation of AC voltage at a specific frequency, the vertical vibration of the piezoelectric vibrator induces a net unidirectional flow in the pump chamber. Additionally, the curved design of the CFTs increases the flow area and allows them to serve as alternatives to traditional cooling tubes, reducing the space occupation in cooling systems.

### 2.3. Theoretical Analysis

The CFTVPP consists of a pump body and a PZT vibrator. The upper surface of the pump body features a recessed, flattened cylindrical groove that forms a pump chamber with the PZT vibrator. When the PZT vibrator operates in a bending vibration mode, the pressure changes within the pump chamber cause the fluid inside to undergo a reciprocating flow. The motion of the fluid inside the pump chamber can be divided into two processes, suction and discharge, in accordance with the displacement direction of the PZT vibrator. Given the stable vibration of the PZT vibrator, the volume of fluid intake or discharge can be assumed to be equal for each cycle. Additionally, the fluid density ρ is assumed to be constant. For a PZT vibrator operating in B00 mode, the displacement and the generated pressure at the center can be expressed as follows [39]:

(1) $$H_{0}=\frac{3}{8}\frac{d^{2}}{\delta^{2}}d_{31}U$$

(2) $$P_{g}=\frac{12\pi Y_{11}^{D}d_{31}}{4.5\pi Y_{11}^{D}g_{31}d_{31}+1}\frac{\delta }{d^{2}}U$$

Here, *d* and *δ* represent the diameter and thickness of the PZT vibrator, respectively. Moreover, $d_{31}$ and $g_{31}$ are the piezoelectric constants, and $Y_{11}^{D}$ is the elastic modulus of the piezoelectric material. *U* represents the driving voltage. When a voltage is applied, assuming the displacement of the PZT vibrator follows a circular arc, the change in pump chamber volume can be given by the following equation:

(3) $$\Delta V=\frac{\pi d^{2}}{8}H_{0}=\frac{3\pi }{64}\frac{d^{4}}{\delta^{2}}d_{31}U$$

When the fluid flows through the CFT, the pressure drop Δ*P* can be expressed as

(4) $$\Delta P=\frac{\xi \rho v^{2}}{2}$$

where ρ is the fluid density, v is the average flow velocity of the fluid in the flow tube, and ξ is the flow resistance coefficient.

Given that the radius of the CFT is constant, the volume of fluid flowing through the flow tube can be expressed as

(5) Q=Sv

where *S* is the cross-sectional area of the tube.

**Figure 5:**
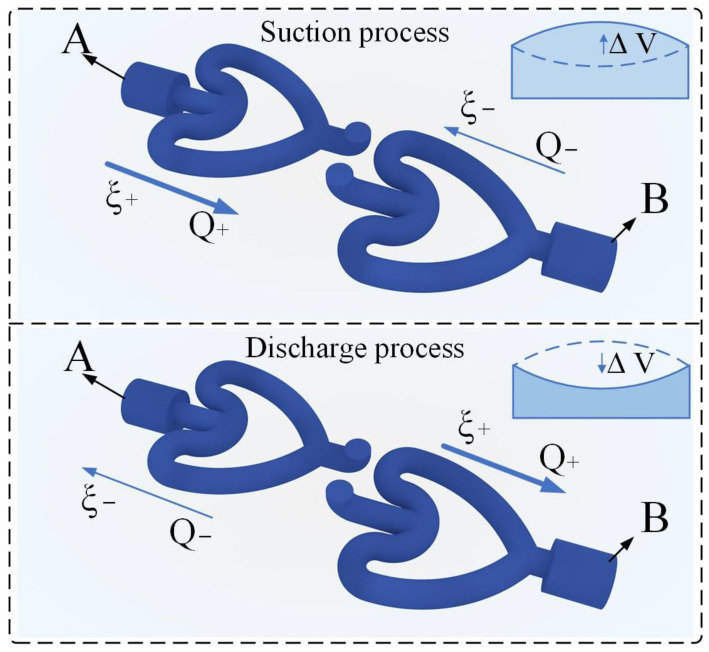
Schematic of the suction and discharge processes of the CFT.

For simplicity, the subscripts “+” and “−” represent the physical quantities during forward and reverse flow processes, respectively. In Figure 5, the blue region represents the flow domain of the CFT. The external pressure of the piezoelectric pump is assumed to be standard atmospheric pressure. Therefore, the pressure drop ∆*P* between the pump chamber and the two external interfaces, A and B, is considered to be equal. In accordance with Equations (4) and (5), the volume of fluid flowing through the tube can be expressed as

(6) $$Q_{+}=S{\left(\frac{2\Delta P}{\rho }\right)}^{1/2}{\left(\frac{1}{\xi_{+}}\right)}^{1/2}=C\xi_{+}{}^{-1/2}$$

(7) $$Q_{-}=S{\left(\frac{2\Delta P}{\rho }\right)}^{1/2}{\left(\frac{1}{\xi_{-}}\right)}^{1/2}=C\xi_{-}{}^{-1/2}$$

(8) $$C=S{\left(\frac{2\Delta P}{\rho }\right)}^{1/2}$$

where $Q_{+}$ represents the instantaneous flow rate during forward flow, and $Q_{-}$ represents the instantaneous flow rate during reverse flow.

When the center of the PZT vibrator moves from the lowest point to the highest point, the pump chamber intakes fluid. The CFT connected to external interface A is in the forward flow state, while the other one is in the reverse flow state. Combining Equations (6) and (7), we have

(9) $$\Delta V=\bar{Q_{+}}+\bar{Q_{-}}=C(\xi_{+}^{-1/2}+\xi_{-}^{-1/2})$$

where $\bar{Q_{+}}$ and $\bar{Q_{-}}$ represent the average values of $Q_{+}$ and $Q_{-}$, respectively, during the period when the center of the PZT vibrator moves from the lowest point to the highest point.

Through solving Equations (3) and (9), *C* can be obtained as follows:

(10) $$C=\frac{\frac{3\pi }{64}\frac{d^{4}}{\delta^{2}}d_{31}U}{\left(\xi_{+}^{-1/2}+\xi_{-}^{-1/2}\right)}$$

When the center of the PZT vibrator moves from the highest point to the lowest point, the pump chamber discharges fluid. Without the effect of fluid pressure on vibration, the amount of fluid discharged by the pump can also be expressed using Equation (9). However, in this case, the direction of fluid flow within the CFT completely changes. The flow tube that was originally in forward flow becomes reverse flow, and vice versa. Therefore, the total pump flow can be expressed as the difference in the flow rates of any single CFT, denoted as Δ*V*′, i.e.,

(11) $$\Delta V\prime =Q_{+}-Q_{-}=C\left(\xi_{+}^{-1/2}-\xi_{-}^{-1/2}\right)$$

Given that each flow tube undergoes only one transformation in a single cycle, the flow rate per unit time at a vibration frequency of *f* can be expressed as

(12) $$V=f\Delta V\prime =\frac{3\pi }{128}\frac{d^{4}}{\delta^{2}}d_{31}Uf\frac{\eta -1}{\eta +1}$$

where $\eta ={\left(\frac{\xi_{-}}{\xi_{+}}\right)}^{1/2}$, and $\xi_{+}$ and $\xi_{-}$ are the flow resistance coefficients for forward and reverse flow, respectively. These coefficients are difficult to obtain analytically, but they can be determined using the finite element method by evaluating the pressure loss and average velocity at the inlet and outlet. They are then substituted into Equations (9) and (10) for calculation.

## 3. Simulation Analysis

The energy loss and pressure drop of the cardioid flow tube were analyzed by simulating the fluid flow within the flow tube. The modeling software used was Solidworks (Solidworks 2023, Dassault Systemes S.A., Waltham, MA, USA), and the model was then imported into ANSYS (ANSYS 2022 R1, Swanson Analysis Systems, Inc., Canonsburg, PA, USA) to create boundary names and generate the mesh based on the turbulence model. To ensure more accurate simulation results, the cross-section of the cardioid flow tube was refined. Before confirming the mesh refinement, a grid independence verification was performed. For this purpose, the pressure loss across the five sets of CFTs was used as an indicator to assess the influence of mesh refinement on the calculation results. The realizable k-epsilon [40] turbulence model was applied to accurately predict the flow in the curved tubes of the CFTs, which consists of the transport equations for turbulent kinetic energy and turbulent dissipation rate.

(13) $$\frac{\partial \left(\rho K\right)}{\partial t}+\frac{\partial \left(\rho \bar{u_{j}}K\right)}{\partial x_{j}}=\frac{\partial }{\partial x_{j}}\left[\left(\mu +\frac{\mu_{t}}{{\mathrm{Pr}}_{K}}\right)\frac{\partial K}{\partial x_{j}}\right]+P_{K}+G_{b}-\rho \varepsilon -Y_{M}$$

(14) $$\frac{\partial \left(\rho \varepsilon \right)}{\partial t}+\frac{\partial \left(\rho \bar{u_{j}}\varepsilon \right)}{\partial x_{j}}=\frac{\partial }{\partial x_{j}}\left[\left(\mu +\frac{\mu_{t}}{{\mathrm{Pr}}_{\varepsilon }}\right)\frac{\partial \varepsilon }{\partial x_{j}}\right]+\rho C_{1}\bar{S}\varepsilon -C_{2}\rho \frac{\varepsilon^{2}}{K+\sqrt{\nu \varepsilon }}+C_{\varepsilon 1}\frac{\varepsilon }{K}C_{\varepsilon 3}G_{b}$$

In these equations, ρ and μ represent the fluid density and dynamic viscosity, *K* is the turbulent kinetic energy, $u_{j}$ is the velocity vector, $x_{j}$ is the spatial coordinate, $P_{K}$ is the transport term of turbulent kinetic energy, *ε* is the turbulent dissipation rate, $\mu_{t}$ is the turbulent viscosity, and $C_{\varepsilon 1}$ and $C_{\varepsilon 3}$ are empirical constants. For boundary conditions, the inlet was set as a pressure inlet with a total pressure of 3000 Pa, while the outlet was a static pressure outlet. The turbulence intensities were set at 1% for both forward and reverse flow. The numerical convergence criterion was set to 10^−5^ to enhance the calculation accuracy. The solution scheme used was the SIMPLE scheme, and a second-order upwind scheme was applied for the discretization of the equations. Considering the scale of the flow tube, the wall condition was set at a roughness constant of 0.5 for a non-slip stationary wall. Table 3 provides the results of the grid independence calculation. When the grid quantities for the four sets of CFTs reached 2,634,017, 2,743,472, 3,497,879, 2,873,943, and 2,898,564, the error was less than 0.4%. Therefore, these grid quantities were chosen as the solution in the simulations.

**Figure 6:**
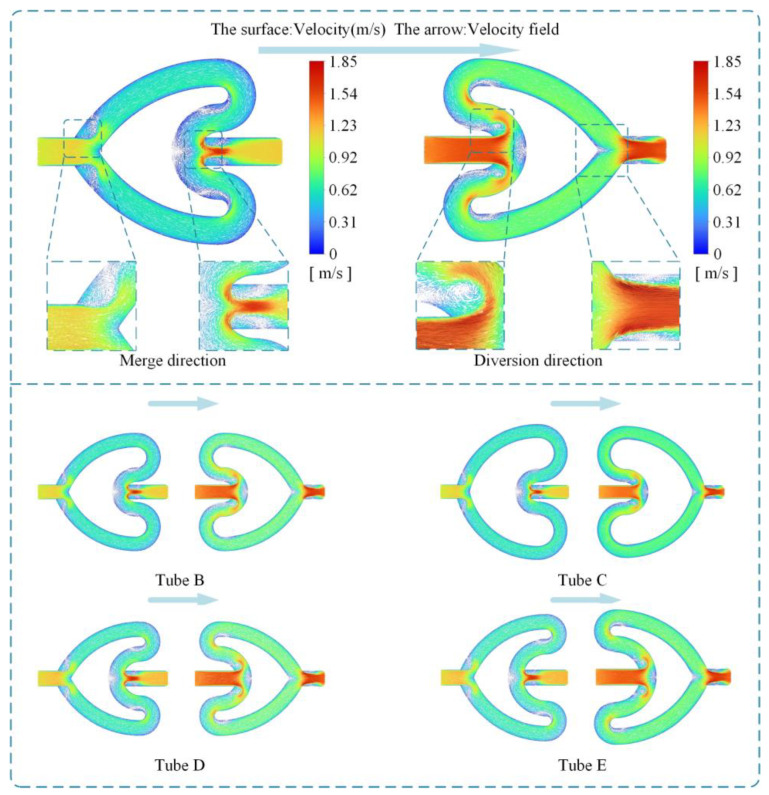
Velocity vectors in the X–Y plane of the CFT.

**Figure 7:**
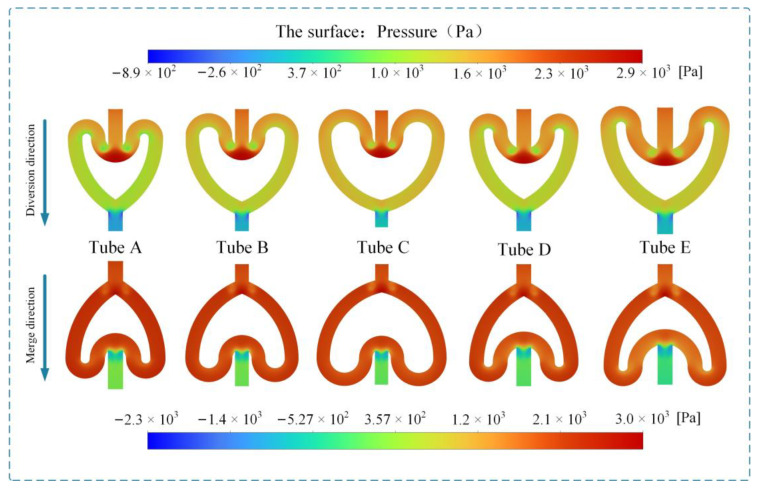
Pressure contour in the X–Y plane of the CFT.

Figure 6 shows the velocity vector maps of the five CFTs during forward and reverse flows. In the reverse flow process, vortices appear at the edges of the convex and concave bifurcation points, with larger vortices at the edges of the convex bifurcation point. This result indicates that the fluid dissipates some mechanical energy, resulting in pressure loss. In the forward flow process, the pressure values at the concave bifurcation point are the highest, which is due to the fluid–solid collision caused by inertia. After passing through the curved tubes on both sides, the flow losses are manifested as a decrease in the total pressure, leading to friction losses and secondary flow losses. Subsequently, the fluid merges into the outlet tube, where no significant vortex phenomenon is observed. The pressure contour plot further illustrates the pressure drop in both forward and reverse flow in the cardioid flow tube, as shown in Figure 7. In forward flow, the pressure exhibits a stepped change, and low-pressure regions appear near the vicinity of the concave bifurcation, providing centripetal force for the vortices. In reverse flow, there is no significant pressure change as the fluid enters the concave bifurcation from the inlet. However, when the flow from the bifurcated channels converges at the concave bifurcation, a significant energy loss occurs due to the opposing flow directions, resulting in a larger pressure drop. This is the main reason for the higher pressure drop in reverse flow compared to forward flow.

**Figure 8:**
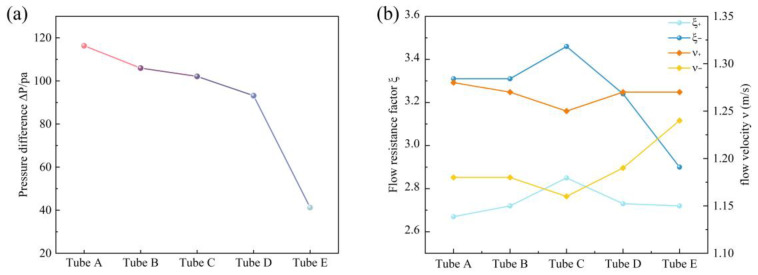
(**a**) Difference in pressure drop for forward and reverse flows of five CFTs under the same boundary conditions; (**b**) flow resistance coefficients and average flow velocities of different flow tubes in the forward and reverse directions in the simulation results.

Under the same boundary conditions, the unidirectionality of the five CFTs was evaluated by comparing the difference in pressure drop between forward and reverse flows. The pressure difference drop for the forward and reverse flows of the five flow tubes is shown in Figure 8a. CFT A has the largest difference between forward and reverse pressure drops, at 116.31 Pa, whereas CFT E has the smallest difference, at only 41.21 Pa. That is, CFT A exhibits the most significant unidirectional flow resistance, and it can achieve optimal pumping performance in the CFTVPP. In accordance with Equation (4), the flow resistance coefficients and average flow velocities for forward and reverse flows obtained from the simulation are shown in Figure 8b. The flow resistance coefficients of CFTs B and C, which have larger radii of curvature in the branching tubes, are noticeably higher than those of the other three tubes. However, the increase in the radius of curvature in the merging tubes has minimal impact on the flow resistance coefficients, and in the case of reverse flow, the flow resistance coefficients of CFTs D and E are even smaller.

## 4. Experimental Setup

### 4.1. Modal Analysis and Amplitude Measurement of PZT Vibrator

**Figure 9:**
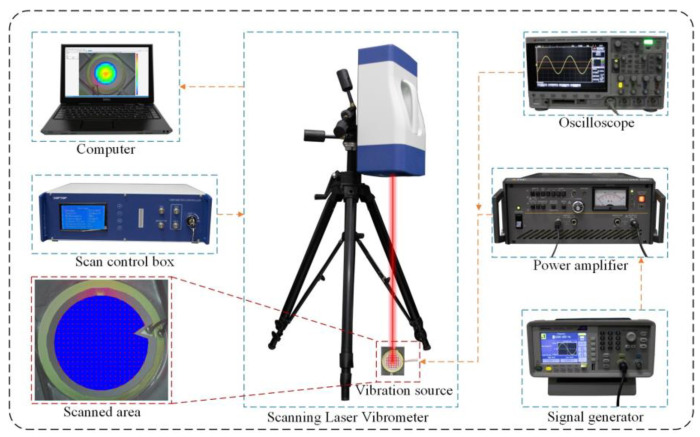
Experimental setup for surface amplitude measurement of PZT Vibrator.

Modal analysis can provide insights into the vibration characteristics of the pumping system at different frequencies. It helps determine the natural frequencies, mode shapes, and corresponding modal forms of the PZT vibrator, aiding in the evaluation and optimization of system performance. Laser Doppler vibrometry was used to measure the displacement of the PZT vibrator. In consideration of the relatively large vibration area of the vibration source and connecting body, a 3D scanning laser vibrometer (LV-SC400, SOPTOP, Yuyao, China) was utilized for displacement measurement. The experimental setup is shown in Figure 9. During the modal test experiment of the PZT vibrator using the scanning laser vibrometer, a three-way joint was connected to the output port of the signal generator, allowing the drive signal of the sample pump to be input into the scanning laser vibrometer. This connection enabled the scanning laser vibrometer to synchronize the vibration data acquisition from different measurement points, starting from the trigger point of the signal. The signal generator was set to output a frequency sweep signal, starting at 10 Hz and ending at 250 Hz, with a scanning time of 1 s. Additionally, the velocity range of the laser vibrometer should be set to 100 mm/s/V, and a low-pass filter of 500 Hz should be applied to avoid distortion of the vibration signal. For the amplitude measurement experiment of the PZT vibrator, the output signal of the signal generator was transformed into a continuous sine wave signal with a frequency of 25 Hz, and the signal was amplified to an effective voltage of 100 V by using a power amplifier. A total of 120 points were uniformly marked on the surface of the PZT vibrator, and the deformation of the entire vibrator was obtained from the displacement data of these 120 points. It is important to note that a reflective film should be attached to the piezoelectric ceramic. One side of the reflective film has a thin adhesive layer, which can adhere to the vibrating surface of the piezoelectric transducer. The other side contains numerous small glass bead particles, which increase the scattering intensity of the laser and facilitate the capture of vibration information. The amplitude results would be displayed on the monitor screen after measurements were taken using the mainframe, and they would change with different excitation voltages and frequencies, which were adjusted using the signal generator and power amplifier. In the amplitude test of the PZT vibrator, deionized water was injected into the CFTVPP by using a syringe to eliminate the influence of internal bubbles on the experiment. In both experiments, an oscilloscope was used to monitor the actual operating voltage, current, and frequency of the PZT vibrator.

### 4.2. Experimental Setup for Characterizing the Output Characteristics of CFTVPP

**Figure 10:**
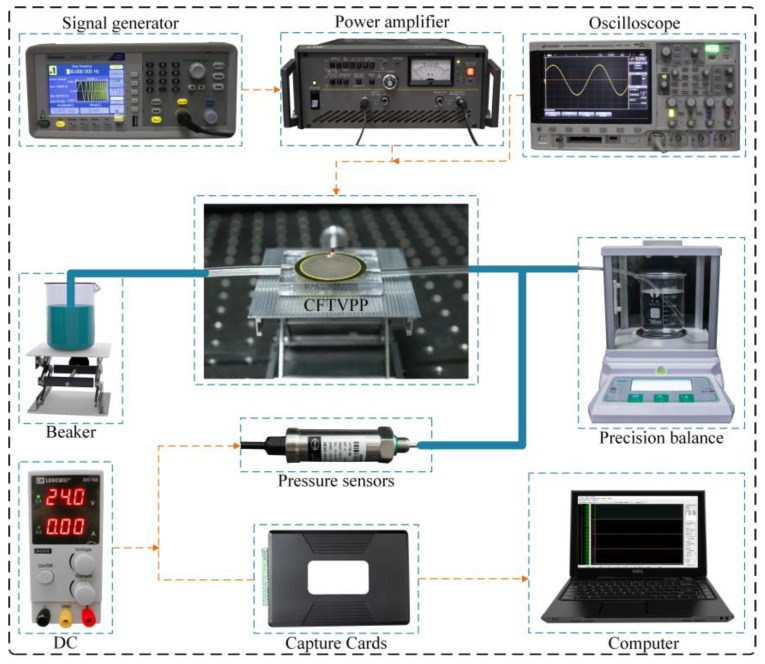
Flow experiment of CFTVPP.

Figure 10 depicts the flow measurement experimental systems for the five sets of CFTVPPs with different parameters. To eliminate the influence of pressure variations on the CFTVPP’s output performance, performance tests were conducted under initial conditions of zero back pressure, and deionized water was used as the working medium to minimize bubble generation. The signal generator provided a sinusoidal signal, which was amplified by the power amplifier and applied to the PZT vibrator. The CFTVPP’s output flow rate per unit of time was measured using an analytical balance (ME204E, Mettler-Toledo, Zurich, Switzerland) with a precision of 0.001 g. The outlet was connected to a dynamic pressure sensor via a T-shaped connector, and the output pressure was measured three times. We measured the effects of voltage and frequency on the output flow rate of the CFTVPP to evaluate the output performance of the five sets of CFTVPPs.

## 5. Results and Discussion

**Figure 11:**
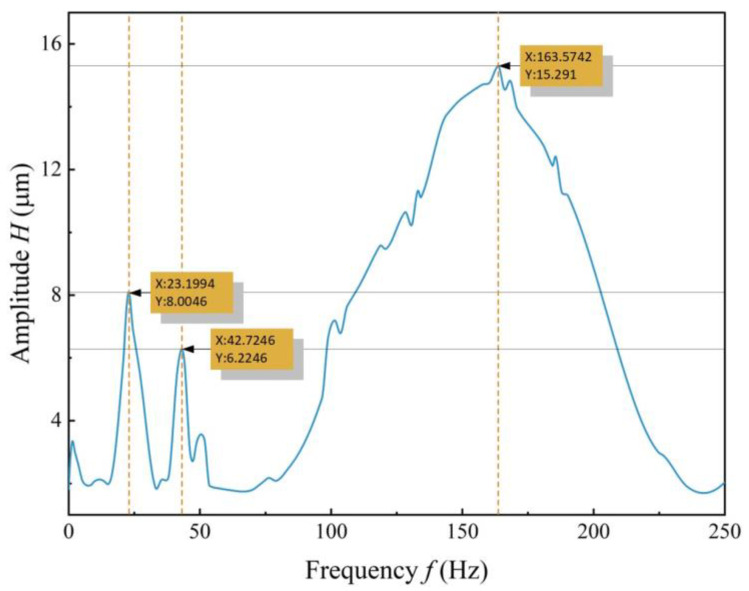
Frequency response curve of the piezoelectric oscillator.

**Figure 12:**
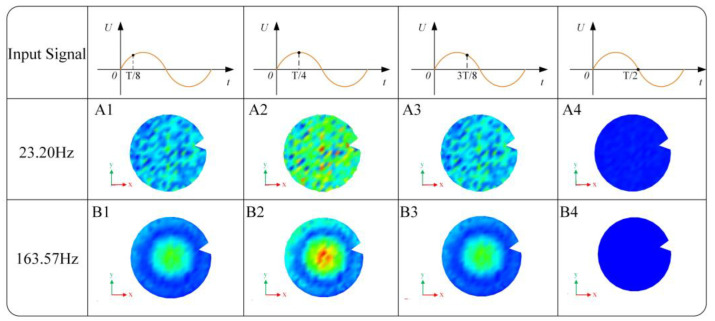
Animation of the vibration pattern of the piezoelectric oscillator at 23.20 and 163.57 Hz frequencies.

Analysis was performed on the software by summing the vibration velocity data of various measurement points obtained from the collected displacement data. The frequency response curve of the PZT vibrator was obtained through a fast Fourier transformation of the velocity data. As shown in Figure 11, three peaks appear in the frequency response curve of the PZT vibrator within the range of 11–500 Hz. The first peak frequency is 23.20 Hz, the second peak frequency is 42.72 Hz, and the third peak frequency is 167 Hz. Figure 12 displays the animation of the vibration modes corresponding to the first and third peak frequencies. The vibration mode of the PZT vibrator around 23 Hz is approximately the B00 mode, while the vibration mode around 163 Hz exhibits a concentric nodal circle corresponding to the B10 mode.

**Figure 13:**
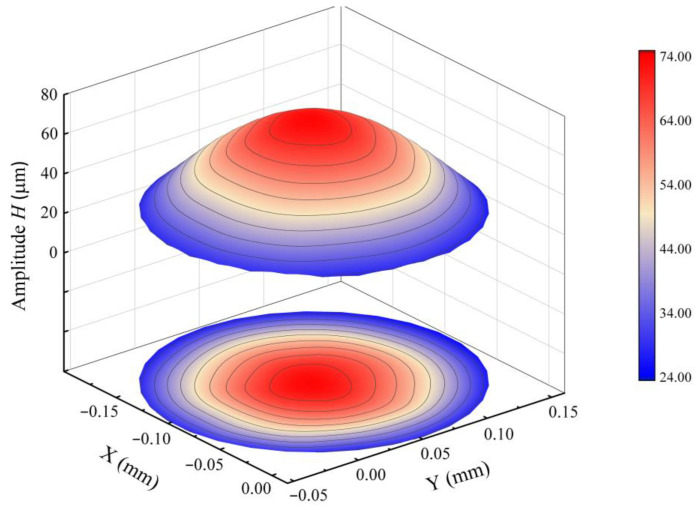
Schematic of the vibrational displacement of the PZT Vibrator.

Figure 13 presents a schematic of the vibration displacement on the surface of the PZT vibrator at a frequency of 23 Hz. The maximum displacement occurs near the center of the vibrator and gradually decreases radially outward. The displacement at various measurement points ranges from 20 μm to 80 μm, and the deformed surface approximates a rotating circular arc.

**Figure 14:**
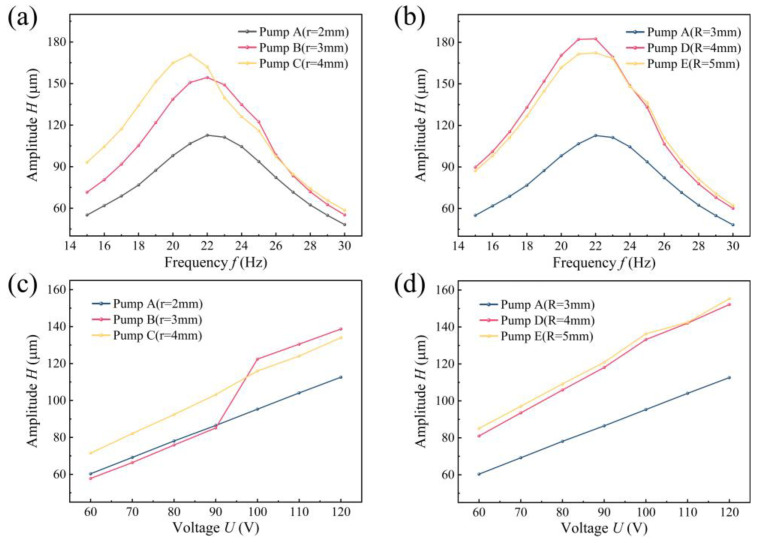
Amplitude characteristics of piezoelectric oscillators for different pumps: (**a**) Amplitude versus frequency for pumps with different radii of curvature R at a fixed voltage (100 V); (**b**) amplitude versus frequency for pumps with different radii of curvature R at a fixed voltage (100 V); (**c**) amplitude versus voltage for pumps with different radii of curvature R at a fixed frequency (25 Hz); (**d**) amplitude versus voltage for pumps with a fixed frequency (25 Hz) of the amplitude versus voltage for pumps with different radii of curvature R.

Figure 14 presents the amplitude measurement results for different pumps. At a fixed AC voltage of 100 V, the amplitude of the PZT vibrator for each pump increases and then decreases with frequency. Among them, the CFTVPP with the largest curvature radius in the branching tube, CFTVPP D, exhibits the highest amplitude at 22 Hz, reaching 182.5 μm. Except for CFTVPP C, the maximum amplitudes for the other pumps occur at 22 Hz. CFTVPP A has a maximum amplitude of 112.7 μm, CFTVPP B has a maximum amplitude of 154.4 μm, CFTVPP E has a maximum amplitude of 172.4 μm, and CFTVPP C has a maximum amplitude of 170.8 μm at 21 Hz. These results indicate that the amplitude changes of the oscillators vary linearly with the voltage and are similar to the trend in flow rate and amplitude curves.

**Figure 15:**
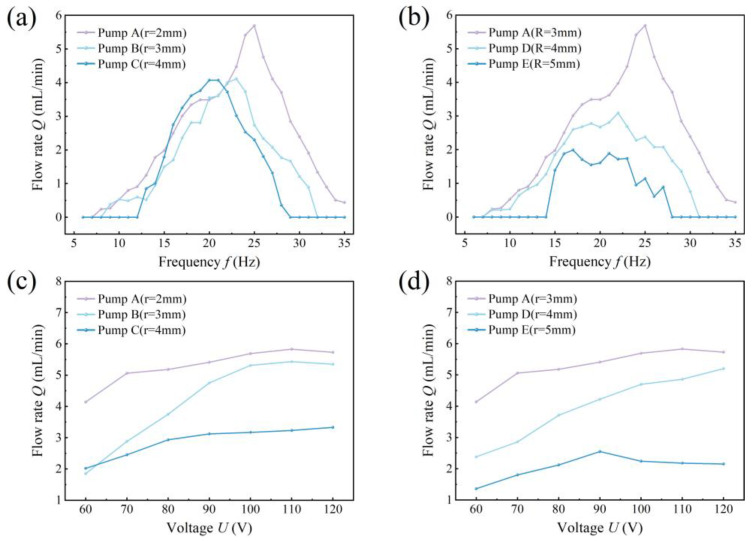
Net flow rate measurements for different pumps. (**a**) Net flow rate of pumps with different radii of curvature r at fixed voltage; (**b**) net flow rate of pumps with different radii of curvature R at fixed voltage; (**c**) net flow rate of pumps with different radii of curvature r of the merging bend at fixed frequency; (**d**) net flow rate of pumps with different radii of curvature R at a fixed frequency.

Figure 15 compares the output flow rates of the different pumps per minute. The output flow rates of the cardioid pumps vary approximately in a parabolic shape concerning the frequency. Specifically, CFTVPP A achieves the highest output flow rate of 5.96 mL/min at a frequency of 25 Hz, CFTVPP B reaches the maximum output flow rate of 4.11 mL/min at a frequency of 23 Hz, CFTVPP C obtains the maximum output flow rate of 4.07 mL/min at a frequency of 21 Hz, CFTVPP D achieves the highest output flow rate of 3.09 mL/min at a frequency of 22 Hz, and CFTVPP E attains the maximum output flow rate of 1.99 mL/min at a frequency of 17 Hz. A comparison of the output flow rates of the CFTVPPs with different curvature radii reveals insignificant differences in pump performance among the different split bending tubes. Meanwhile, the pump performance among the different convergence bending tubes decreases with the increase in curvature radius, which is consistent with the results of the simulations.

**Figure 16:**
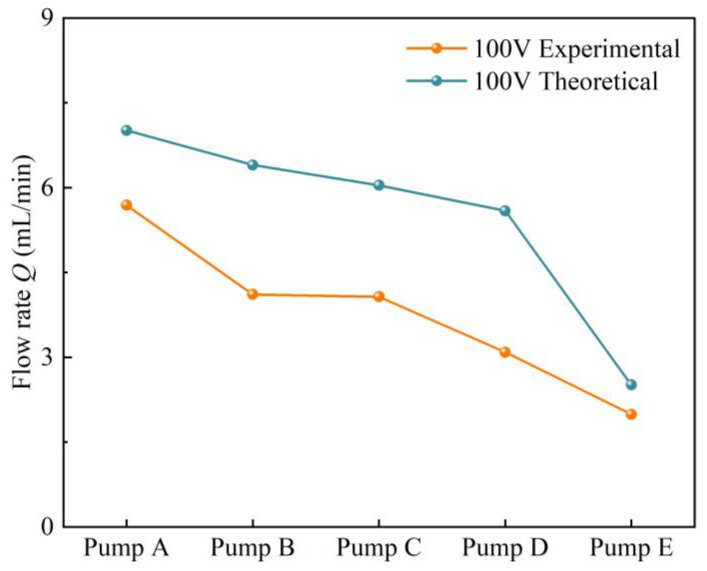
Theoretical and experimental flow rates for different pumps at 100 V.

Table 4 shows the forward and reverse flow resistance coefficients calculated from the simulation data. Figure 16 compares the experimental flow rates with the calculated theoretical flow rates by substituting the simulated flow resistances into Equation (12). Under the conditions of 100 V and 23 Hz, the maximum theoretical flow rate is 13.98 mL/min, whereas the maximum experimental flow rate is 5.69 mL/min. The experimental and theoretical flow rates show consistent trends among the different pumps, but the theoretical flow rates are higher than the experimental flow rates. The reason for this discrepancy is the simplification of the flow tube wall function. In the simulation, the flow tube walls are assumed to be smooth, non-slip, stationary walls with a roughness constant of 0.5. However, due to the 3D printing process used to fabricate the pump body and CFT components of the CFTVPP, the inner walls of the CFTs are not perfectly smooth due to manufacturing tolerances. Additionally, the piezoelectric transducer is bonded to the pump chamber with silicone adhesive, indirectly increasing the volume of the pump chamber and reducing the variability in its volume to some extent. The presence of bubbles in the pump chamber during the experiment also contributes to the reduced pump flow rate to some degree.

**Figure 17:**
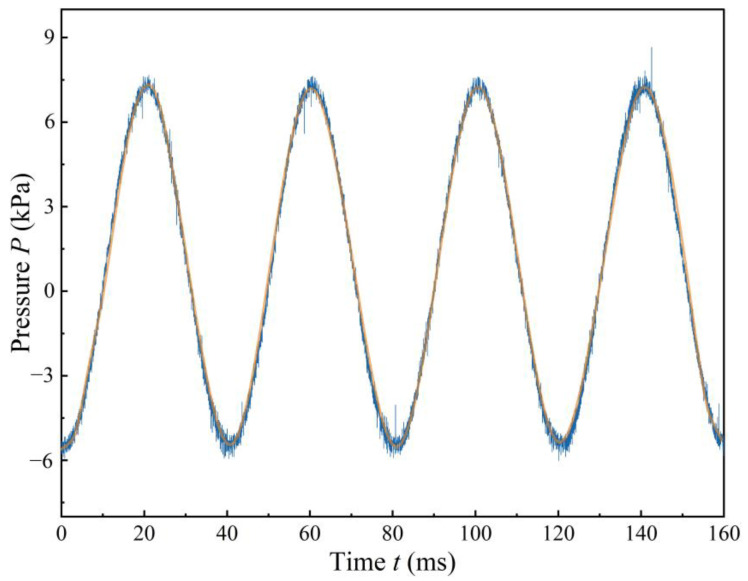
Curve of output pressure of CFTVPP as a function of time.

**Figure 18:**
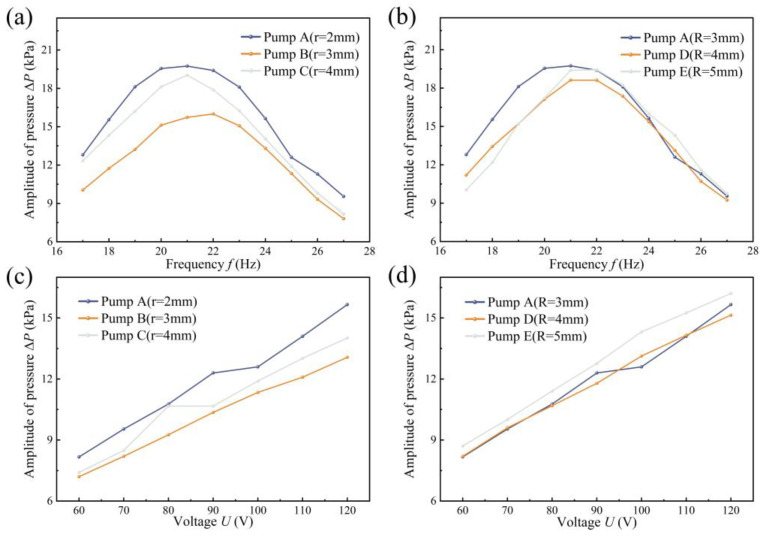
Output pressure measurements of different pumps. (**a**) Output pressure of pumps with different radii of curvature r at fixed frequency; (**b**) output pressure of pumps with different radii of curvature R at fixed frequency; (**c**) output pressure of pumps with different radii of curvature r of merging bends at fixed voltage; (**d**) output pressure of pumps with different radii of curvature R at a fixed voltage.

The variation in pressure measurements with time, as measured by the pressure sensor, is shown in Figure 17. Under the excitation of a sinusoidal signal, the up-and-down vibration of the piezoelectric vibrator causes periodic changes in the volume of the pump chamber, resulting in a pressure–time curve that approximates a smooth sinusoidal waveform. The difference between the maximum and minimum output pressures is defined as the pulsatile pressure amplitude, denoted as Δ*P*, which represents the output pressure measurements of the different pumps. In Figure 18, the pressure variation curves among the different pumps exhibit similar properties, with peaks occurring around a frequency of 22 Hz. Among them, CFTVPP A achieves the highest output pressure of 19.74 kPa. The maximum output pressures of CFTVPP B–E are 16, 19.03, 18.62, and 19.44 kPa, respectively. The output pressures are proportional to the applied voltage, which is consistent with the trends observed for the amplitude and flow rate curves. Based on the comparison among the five different CFTVPPs, the pumps with a curvature radius of 3 mm for the converging bend and a curvature radius of 2 mm for the diverging bend demonstrate superior performance in terms of flow rate. Hence, these specific designs are more suitable for integration into piezoelectric pumps for efficient fluid transportation.

## 6. Conclusions

This study proposed a CFTVPP. The structure and working principle of the flow tube were analyzed, and theoretical models for vibration, fluid dynamics, and net flow rate were established. By controlling the curvature radius parameters of the curved tube, five different CFTs were designed, and the velocity and pressure fields within the tubes were simulated. Numerical calculations were used to evaluate the flow resistance and pressure drop differences between forward and reverse flows for the five tube designs. Additionally, a valveless piezoelectric pump model was fabricated using 3D printing technology. Experimental tests were conducted, and the output performance of the five piezoelectric pumps was compared. The results demonstrated that the size parameters of the merging tube significantly affected the pump’s output performance, with smaller curvature radii resulting in higher output flow rates. Among the five CFTs, the tube with a merging tube curvature radius of 2 mm exhibited the highest efficiency, achieving a maximum net flow rate of 5.69 mL/min. The CFTVPP designed in this study presents a simple overall structure and superior output performance, making it suitable for applications in medical transport, fluid mixing, and cooling systems, among other fields. However, there are still some aspects that deserve further research. Firstly, the paper only discusses the relative performance of the five sets of CFTs in terms of their output characteristics, which provides a reference for future structural optimization work. It would be beneficial to investigate if there are theoretical formulas that can calculate the optimal curvature radius for the curved tubes. Secondly, due to limitations in manufacturing conditions, the CFTs studied in this research have a diameter in the millimeter range. With the emergence of MEMS microfabrication technologies such as deep reactive ion etching and electron beam exposure, the downsizing of microfluidic devices has become much easier. It would be worthwhile to explore the performance of CFTVPPs at the micro and nanoscale in order to accelerate their applications in the fields of chemistry and biomedical sciences.

## Figures and Tables

**Table 1:** Dimensional parameters of the five CFTs.

Category	r	R
Tube A	2 mm	3 mm
Tube B	3 mm	3 mm
Tube C	4 mm	3 mm
Tube D	2 mm	4 mm
Tube E	2 mm	5 mm

**Table 2:** Structural parameters of the PZT Vibrator.

Parameters	Title 3
Resonant frequency (kHz)	2.6 ± 0.5
Resonant impedance (Ω)	<250
Free capacitance (pF)	350,000 ± 30%
Plate material	Brass
Plate diameter (mm)	41 ± 0.1
Plate thickness (mm)	0.25
Plate density (kg/m^3^)	8.5 × 103
Ceramic disc diameter (mm)	35 ± 0.2
Ceramic disc thickness (mm)	0.25
Ceramic disc density (mm)	7.5 × 103
Total weight (g)	4.52

**Table 3:** Grid independence verification.

Category	N/10^6^	∆P/Pa	*r*/%
Tube A	1.76	2203.79	
	2.18	2192.91	0.49
	2.63	2186.41	0.30
	3.14	2184.43	0.09
Tube B	1.88	2206.78	
	2.30	2197.57	0.42
	2.74	2192.37	0.24
	3.22	2191.04	0.06
Tube C	2.57	2236.19	
	3.09	2225.87	0.46
	3.50	2219.46	0.29
	3.92	2217.52	0.09
Tube D	1.92	2215.66	
	2.47	2204.75	0.49
	2.87	2198.86	0.27
	3.36	2197.14	0.08
Tube E	2.01	2203.59	
	2.43	2193.13	0.48
	2.90	2187.80	0.24
	3.34	2186.70	0.05

**Table 4:** Flow resistance coefficients obtained by simulation.

Category	Forward Flow Resistance	Reverse Flow Resistance
Tube A	2.67	3.31
Tube B	2.72	3.31
Tube C	2.85	3.46
Tube D	2.73	3.24
Tube E	2.72	2.90

## Data Availability

The datasets generated or analyzed during the current study are available from the corresponding author upon reasonable request.
